# An Online Mindfulness Intervention for International Students: A Randomized Controlled Feasibility Trial

**DOI:** 10.32872/cpe.9341

**Published:** 2023-06-29

**Authors:** Sumeyye Balci, Ann-Marie Küchler, David Daniel Ebert, Harald Baumeister

**Affiliations:** 1 Department of Clinical Psychology and Psychotherapy, Institute of Psychology and Education, Ulm University, Ulm, Germany; 2 Department of Sport and Health Sciences, Technical University of Munich, Munich, Germany; Philipps-University of Marburg, Marburg, Germany

**Keywords:** e-health, digital health, student mental health, cultural adaptation, internet intervention, international student

## Abstract

**Background:**

Student mobility across borders poses challenges to health systems at the university and country levels. International students suffer from stress more than their local peers, however, do not seek help or underutilize existing help offers. Some barriers to help-seeking among international students are insufficient information regarding the health offers, stigma, and language, which might be overcome via culturally adapted internet and mobile-based interventions (IMI).

**Method:**

A randomized controlled feasibility trial with a parallel design assessed the feasibility and potential efficacy of an online mindfulness intervention adapted for international university students. Participants were randomized into either an adapted online mindfulness intervention (StudiCareM-E) (IG, n = 20) or a waitlist control group (WL, n = 20). Participants were assessed at baseline (t0) and eight-week post-randomization (t1). The feasibility of StudiCareM-E was evaluated regarding intervention adherence, client satisfaction, and potential negative effects. The potential efficacy of StudiCareM-E was measured by means of the level of mindfulness, perceived stress, depression, anxiety, presenteeism, and wellbeing. Efficacy outcomes were evaluated with regression models on the intention-to-treat (ITT) sample (n = 40), adjusting for the baseline values.

**Results:**

Participants’ formative feedback suggested improvements in the content of the IMI. There were no crucial negative effects compared to WL. Assessment dropout was 35% (IG: 50%: WL: 20%), and intervention dropout was 60%. StudiCareM-E yielded significant improvements in mindfulness (β = .34), well-being (β = .37), and anxiety (β = -.42) compared to WL.

**Conclusion:**

StudiCareM-E might be used among culturally diverse international student populations to improve their well-being. Future studies might carefully inspect the extent of the adaptation needs of their target group and design their interventions accordingly.

## Highlights

- International students suffer from more stress compared to their local peers but rarely seek help.
- Internet interventions can be adapted to cater to the needs of culturally diverse international students.
- The adapted internet intervention for international students offers great potential to improve psychological outcomes.

Starting university after high school is a challenging time. University students experience stress due to financial issues, love life, and family relationships (Karyotaki et al., 2020), and sexual identity (Rentería et al., 2021). Exposure to these stressors might result in developing a mental health problem or low academic functioning, even dropping out of university (Athira et al., 2020; Bantjes et al., 2021; Bruffaerts et al., 2018). Prevalence of mental health problems among university students assessed from eight countries, and 19 universities, resulted in 35% of student participants (*N* = 13.984) having at least one mental health problem (i.e. anxiety, mood, or substance use), with Major Depressive Disorder (MDD) (21.2% lifetime prevalence, 18.5% 12-month prevalence) being the most common and Generalized Anxiety Disorder (GAD) the second most common (18.6% lifetime prevalence, 16.7% 12-month prevalence) (Auerbach et al., 2018). The burden from mental health problems comprises 45% of the overall disease burden among 10-24-year-olds (Gore et al., 2011). Moreover, the majority of mental health problems over the lifetime first develop before the age of 24, which makes this time of university crucial to screen for mental health problems and provide prevention and/or treatment opportunities (Jones, 2013).

Students who cross borders to study are increasing in Europe, especially in Germany, where the number of international students substantially increased from 312.000 in 2018 to 416.437 in 2020 (Eurostat, 2018; Statistisches Bundesamt, 2021). International students encounter similar life challenges as students studying in their home country but are also faced with additional stressors that may trigger homesickness (Akhtar & Kröner-Herwig, 2015), problems in socializing with the local students (Byrne et al., 2019), adapting to a new country, lifestyle, and language, and a new academic culture and customs (Forbes-Mewett & Sawyer, 2016; Yu & Wright, 2016). Studying abroad, while mostly associated with positive experiences, can cause some challenges and result in mental burden (Orygen, 2020; Stokes et al., 2021).

Even though university students suffer from psychological distress, their help-seeking behavior is very limited (Auerbach et al., 2016). This can be attributed to various factors, such as not being familiar with the symptoms of or the help options for mental health problems, social stigma, social and cultural influences (e.g. traditional masculine ideals) (Lynch et al., 2018), limited access to professional help via university, and financial problems (Auerbach et al., 2016; Gulliver et al., 2010; Orygen, 2020). Although their psychological stress level is higher compared to students of the host country (Lu et al., 2014), international students are less likely to seek help from a counseling service (Lu et al., 2014; Stokes et al., 2021), have lower mental health literacy, and less positive attitudes towards seeking help (Clough et al., 2019). Some barriers which are specific to international students might be related to cultural backgrounds where symptom severity is underestimated, hesitation because of their family’s reaction, and language barrier (Lu et al., 2014). In general, cultural influences play an important role in attitudes toward mental health and help-seeking (Hudak et al., 2018). Furthermore, international students who reach out to a counseling service fail to utilize psychological help services, e.g. not attending the necessary number of sessions, and even benefit less from it, compared to local students who utilized these services (Stokes et al., 2021), and drop out of the treatment prematurely (Nilsson et al., 2004). In summary, there is a persistent discrepancy between mental health needs and actual help-seeking behavior among international students. Therefore, it is critical to offer appropriate psychological help to this particularly vulnerable sub-group of the student population (Teegen & Conrad-Popova, 2021).

Barriers to help-seeking could be overcome by an easily accessible offer via delivering psychological health interventions online. Internet- and mobile-based interventions (IMI) have the advantage of being independent of time and place, ability to reach populations otherwise hard to reach, offering interventions to treat and prevent various psychological problems, and are cost-effective (Ebert et al., 2018). Likewise, IMI have proven to be effective in university student populations with small to moderate effects in decreasing psychological symptoms (Harrer et al., 2019). Provided as guided IMI they could work as effectively as face-to-face cognitive behavioral therapy (Carlbring et al., 2018). The limited number of studies that targeted international students’ wellbeing via offering an IMI resulted in improved mental health (Kanekar et al., 2010), reduction of sleep difficulties (Spanhel, Burdach, et al., 2021), more help-seeking, and reduced stigma (Clough et al., 2020). However, issues around the adherence and uptake of IMI still persist (Batterham et al., 2021; Molloy et al., 2021). IMI can also aim at treating mental health problems, e.g. depression, but can also be utilized in promoting health skills (Galante et al., 2018; Sevilla-Llewellyn-Jones et al., 2018). An example of a helpful skill to promote mental health and well-being is mindfulness. Mindfulness refers to experiencing the present and being aware of life with acceptance and self-compassion, without any judgment (Slom & Kabat-Zinn, 2020). Mindfulness-based interventions could be delivered successfully online (Jayawardene et al., 2017), and have been tested and found effective among students (Hall et al., 2018; Mak et al., 2015; Nguyen-Feng et al., 2017) and general and clinical populations (Querstret et al., 2018; Sevilla-Llewellyn-Jones et al., 2018). A recent meta-analysis of RCTs of online mindfulness interventions resulted in significant small to moderate effects on depression (*g* = .34), anxiety (*g* = .26), mindfulness (*g* = .40), stress (*g* = .44), well-being (*g* = .22). These effects were maintained in the follow-up for depression (*g* = .25) and anxiety (*g* = .23) (Sommers-Spijkerman et al., 2021). Mindfulness interventions can be seen as less threatening due to their associations with well-being and calmness, instead of interventions targeting mental health problems which might impede help-seeking due to stigma (Clement et al., 2015). Mindfulness interventions could also be adapted to meet the needs of a specific target group. For instance, the delivery method could be changed (e. g. intervention taking place in a cultural community center), the facilitator, researcher/therapist, could be matched with a target group’s cultural background, a culturally congruent recruitment strategy could be adopted, the content could be changed, culturally appropriate analogies could be used (Watson-Singleton et al., 2019), dispelling myths around mindfulness (Castellanos et al., 2020; Cotter & Jones, 2020; Lawlor, 2022), storytelling, and community input can be utilized (Le & Gobert, 2015). However, the adaptation of online mindfulness interventions is rarely defined in detail in the previous literature, but systematic adaptation frameworks are emerging (Loucks et al., 2022; Spanhel, Balci, et al., 2021). Moreover, mindfulness interventions’ transdiagnostic nature and growing popularity in recent years via advertising as a self-care instrument make them more appealing. They could therefore serve as an alternative way to reach out to international students with various psychological problems.

## Objectives

In order to explore the feasibility and possible efficacy of the online mindfulness intervention adapted for international students, StudiCare Mindfulness – English version (StudiCareM-E), the following research questions will be explored.

 

**Research questions:**
1. Are the study methods feasible and transferable to a future, large-scale randomized controlled trial with regard to implementation and the chosen recruitment strategy?
2. What are the levels of intervention satisfaction, adherence, negative effects, and acceptance?
3. Does the internet-based intervention StudiCareM-E have a potential effect on increasing mindfulness levels compared to a waitlist control group?
4. What effects does the StudiCareM-E have on measures of psychological well-being (depression, stress, anxiety, well-being, and presenteeism) in comparison to the waitlist control group?

## Method

This is a two-armed, randomized controlled trial of parallel design (registered in the German Clinical Trials Register DRKS00017507) comparing guided IMI StudiCareM-E (IG) with a waitlist control group (WL) receiving the unguided version of the same IMI eight weeks post-randomization. The study was approved by the ethics committee of Ulm University (Number 413/18) and followed the CONSORT guidelines for feasibility trials (Eldridge et al., 2016).

### Participants

The eligibility criteria for participating in the study were: being at least 18 years old, having a low to moderate level of mindfulness (Freiburg Mindfulness Inventory FMI < 37), having internet access, having student status, ability to read and understand English (all self-reported), giving consent to participate in the study. Exclusion criteria included being in a mindfulness course, having a higher than moderate mindfulness level, and being in psychotherapy.

### Procedure

Participants were recruited from July 2019 to March 2020. The recruitment was done through regular emails sent out twice a year from the cooperating universities of the StudiCare project (Harrer et al., 2018; Küchler et al., 2019) in Germany, Switzerland, and Austria, complemented by study posters and further on-site recruitment strategies at the Ulm University. The email consisted of information regarding various trainings that are offered within the StudiCare project at a given time along with an invitation to participate in the training. Additional emails were sent to universities’ international offices in the above-mentioned countries. Potential participants received a direct link to the study website to register and were then invited to the screening via email. After screening and providing informed consent, participants were invited to complete the initial survey. Participants were randomized into either intervention (immediate access) or waitlist (access eight weeks post-randomization) control group. Afterward, they got access to online training.

### Randomization

Randomization was carried out by an independent researcher who was not involved in the Studicare Project. A simple randomization list applying block sizes of two and four by a computer generator was created using Sealed Envelope^1^1https://www.sealedenvelope.com/simple-randomiser/v1/lists. 20 participants were allocated to each study arm with a 1:1 ratio, making a total of 40 participants.

### Intervention

Based on Acceptance and Commitment Therapy (Hayes et al., 1999) and stress management principles (Kaluza, 2015), StudiCareM-E consists of seven weekly modules and two booster sessions; each module takes approximately 50 minutes to complete (Küchler et al., 2020; Schultchen et al., 2020). StudiCareM-E has been shown to yield a high effect among German-speaking students compared to a waitlist control group (*d* = 1.37) (Küchler et al., 2022).

Participants were advised to complete one module per week. Participants who completed seven modules received access to booster sessions one and two, four and 12 weeks, respectively, after completion of the last module. The focus of the intervention is on promoting mindfulness and psychological flexibility. The content is delivered on a content management platform (www.minddistrict.com) via text, images, audio files, and interactive quizzes. Participants were able to access the online platform Minddistrict at all times. Every module aims at improving a different skill, such as identifying stress-inducing thinking patterns and getting in touch with values in life. At the end of each module, homework is assigned to the participant, and at the beginning of the next module, the participants are encouraged to monitor their progress. Each module introduces a different kind of meditation exercise, e.g. body scan, interoception. A mindfulness journal and a summary of the respective module were available at the end of each module. Content and introduced mindfulness exercises of each module are presented in Table 1.

**Table 1:** Intervention Modules and Mindfulness Exercises

Module names	Content	Mindfulness meditation exercises
*Awareness*	An introduction to the concept of mindfulness	Body scan, mindful walking exercise
*Mindful body perception*	Mindful perception of bodily signals	Heart meditation, mindful perception of satiety and hunger
*Stress-aggravating thought*	Mindful coping strategies to deal with stress and distancing from stressful thoughts	Power of thoughts, mindful straightening the posture
*A beneficial thought*	Developing a beneficial thought to deal with stress	Inhaling the beneficial thought, short breathing meditation
*Values in life*	Discovering what is important and valuable in life	Here and now exercise
*Self-care*	Looking at yourself with a loving gaze	Loving and kindness meditation
*Body&mind*	Enjoying small things in life with mindfulness	Shavasana and mindful yoga
*Refresh I&II*	Review of previous modules	Repeating the previous exercises

### Adaptation of the Intervention

Cultural adaptation of the intervention was based on Resnicow’s theory of cultural sensitivity in health behavior intervention development, which has two dimensions: surface and deep structure. According to the theory, interventions could be altered to fit the target groups’ needs and features in these levels where surface-level alterations concern visible characteristics of the target population such as language, music, food choices, and clothing, whereas deep structure changes refer to counting intersecting effects of cultural, social, historical and psychological influences on the target health behavior (Resnicow et al., 2000). In this trial, surface structure changes were conducted to make the intervention content more compatible with culturally diverse international students. Conducted changes to the original German intervention represented in Table 2 based on Spanhel et al.’s taxonomy of cultural adaptation of IMI for mental health problems (Spanhel, Balci, et al., 2021). The taxonomy consists of various components that researchers can adapt in order to make IMI more appropriate to the new target group: ten components related to the content of the intervention, four methodological, and three procedural components. Changes were implemented in content components (e.g. stigmatization of mental health problems), methodological (e.g. guidance in English), and procedural domains (e.g. using a theoretical framework for adaptation). For English-speaking international students, the intervention content of StudiCare-Mindfulness (Küchler et al., 2020; Schultchen et al., 2020) was translated to English and certain aspects (e.g. language barrier, different education systems) changed in accordance with student life and stress sources.

**Table 2:** Culturally Adapted Elements of StudiCare Mindfulness-E

Core components / Specific components	Example
Content components	
*1. Illustrated characters*	
Appearances/ names of characters	change of names of characters to diverse names (e.g. Hua, Andrew, Farah)
Content/ stories/ background of characters	added characters from various regions of the world who migrated to study in Germany
*2. Illustrated activities*	
Daily life	walking the dog, tutoring a fellow student, and contact with family members living abroad
*3. Illustrated environment/ burdens*	
Burdens	high level of pressure for academic excellence, adapting to a foreign academic culture
*4. Language translation*	
Translating intervention	German to English
*5. Language tailoring*	
Simplify text: shortening text passages, simplifying sentences	less technical phrasing, modify wording for easier readability
Use of concrete terms or informal language	the colloquial form was used
Milder descriptions of mental health concepts	describing psychological problems in a university context
*6. Difference in concepts of mental health and its treatment*	
Stigmatization of mental health problems	framing the goal of the intervention as a mindfulness-based stress management tool instead of mental health intervention in order to reduce the stigma
*7. Goals of treatment*	
Increase understanding of treatment possibilities	Introducing various ways of coping with university-related stressors.
*8. Methods of treatment*	
Information/ links to other helpful addresses	psychological help offers which might be available in English are presented to each participant
Methodological components	
*9. Guidance*	
Person used as guide	Guidance by an English-speaking psychologist (SB)
Format of guidance (tailored feedback)	participants can ask for personal contact in addition to semi-structured feedback
Procedural components	
*10. Methods used to obtain information*	
Personal interaction (focus groups, interviews, discussions, think-aloud)	received feedback in the form of qualitative data for the process evaluation and further implementation of the program
Surveys/ questionnaires	assessed acceptance and effectiveness
Pilot/ feasibility studies	this trial has been conducted to measure the feasibility to inform a future definitive trial.
*11. Persons involved*	
Target group and associated people	International students
Professionals working with the target group	International office workers of partner universities distributed recruitment emails
*12. Theoretical framework*	
Guideline for cultural adaptation of face-to-face treatment	surface structure changes were based on the cultural sensitivity framework by Resnicow (Resnicow et al., 2000)

### Guidance

At the end of each module, intervention group (IG) participants received feedback from an e-coach, who was a trained psychologist (SB). Each feedback consisted of a review of their progress in the intervention and encouragement to continue the intervention such as “Dear …., thanks for sending your third module! I am happy that you are working actively on the program.” and continues with a review of completed exercises “The second task was to think about stressful situations in the past and what helped you to cope with stress. You wrote that … was very helpful for you.” and end with an encouragement to continue with the upcoming module “I wish you a relaxed week with many attentive moments and a lot of fun while working on module 4.”. Moreover, reminder emails were sent to the participants who did not complete the modules in time. The e-coach was instructed to take no longer than 15 minutes per feedback, which results in a planned e-coaching time of max. 105 minutes per participant for all seven modules.

### SMS Coach

In IMI, receiving SMS messages may contribute to adherence and intervention effect (Lentferink et al., 2017; Webb et al., 2010). Consequently, a voluntary text message coach was implemented and offered to each participant. These motivational SMS messages were set to be sent every two days, throughout the intervention. They consisted of motivational texts to promote the use of learned skills, be mindful throughout the day, and continue the intervention, such as “‘The true art of life is to see beauty in the daily.’ What beautiful moment did you experience today?”, and “‘Every moment is absolute, alive and meaningful.’ – What was your mindful moment today? When was the least mindful moment? How did you feel then?”.

### Control Group

Control group participants received a document summarizing the alternative support offers via email after the randomization. Participants of the control group got access to the unguided version of the StudiCareM-E eight weeks after the randomization.

### Assessment and Outcomes

Assessments were conducted via an online platform, www.unipark.de, at baseline (t0) and eight weeks post-randomization (t1), blinding of outcome assessment was not possible. All data were self-reported.

Acceptability was measured via participants’ attitudes towards the IMI, their formative feedback, and satisfaction with the intervention and its potential negative effects. Open-ended questions at the end of each module were extracted from the Minddistrict platform. These outcomes are reported descriptively.

The primary efficacy outcome of this study is Mindfulness level. Secondary outcomes are Anxiety, Stress, Depression, Personality, Well-being, Presenteeism, Client Satisfaction, Risks and Negative Effects of Psychotherapy, and Acceptance and Adherence questions.

Mindfulness was assessed using the Freiburg Mindfulness Inventory (FMI), which consists of 14 items measuring mindfulness on a 4-point scale ranging from 1= rarely to 4 = almost always, and showed high internal consistency (α = 0.84) (Walach et al., 2006).

Anxiety was measured with a 7-item Generalized Anxiety Disorder Questionnaire (GAD-7) on a scale from 0 = not at all to 3 = nearly every day and has high internal consistency (α = 0.92) (Spitzer et al., 2006).

Stress outcome was measured with 4-item Perceived Stress Scale (0 = never to 4 = very often), which also showed good reliability (α = 0.77) (Warttig et al., 2013).

Depression was measured with an 8-item Patient Health Questionnaire, where high reliability was observed (α = 0.89) and rated on a scale of 0 = not at all to 3 = early every day (Kroenke et al., 2001).

WHO-5 well-being index was used to assess subjective well-being on a scale of 0 = at no time to 5 = all of the time, which showed high internal consistency, α > 0.80 (Lara-Cabrera et al., 2022; Spanhel, Burdach, et al., 2021; Topp et al., 2015).

Presenteeism, i.e. loss of productivity was measured with the Presenteeism Scale for Students. The subscale of work impairment was used to assess the degree of presenteeism, which consist of 10 items; with total scores ranging from 10 to 50, higher scores represent a higher degree of presenteeism and showed high reliability, α = 0.90 (Matsushita et al., 2011).

Eight weeks after randomization, in addition to the above-mentioned tools, assessments of intervention satisfaction were done using the Client Satisfaction Questionnaire (total scores range from 8 to 32) adapted to Internet-based Interventions (Boß et al., 2016). Negative effects of Psychotherapy were measured using INEP (Inventory for the Assessment of Negative Effects of Psychotherapy) adapted to online interventions with 22 items describing possible negative effects that may occur during the online intervention and whether they are attributed to the intervention (Ladwig et al., 2014). The results of this scale are presented descriptively.

### Sample Size

In order to determine the sample size for this feasibility trial, we followed the recommendation by Whitehead et al. (2016), resulting in a sample size of 15 participants per trial arm for pilot testing of a potential confirmatory trial with 90% power and two-sided 5% significance. A meta-analysis resulted in an effect size of 0.40 for mindfulness-based IMI, therefore we assumed a higher effect size, i.e. 0.50 because this trial is guided (Sommers-Spijkerman et al., 2021). With the expectation of a 30% dropout, we aimed at reaching a sample size of 40 in total.

### Statistical Analyses

IBM SPSS/version 26 and R Studio were used in statistical analyses with a significance level of α = 0.05. Descriptive statistics (means, *SD*s for continuous outcomes, and percentages for categorical variables) were used to summarize the demographic and feasibility data for study groups. Linear regression models were used to investigate potential group differences, where baseline values were used as covariates in all models (dummy coded predictor: IG = 1). For each outcome, we reported standardized regression coefficients and corresponding 95% CI and adjusted *R*^2^ values.

Data analyses were based on the intention-to-treat principle (ITT). Missing data were imputed based on multivariate imputation by chained equations to create 20 completed datasets with 15 iterations. Predictive mean matching was applied as an imputation model.

## Results

### Feasibility

#### Recruitment and Participants

**Figure 1:**
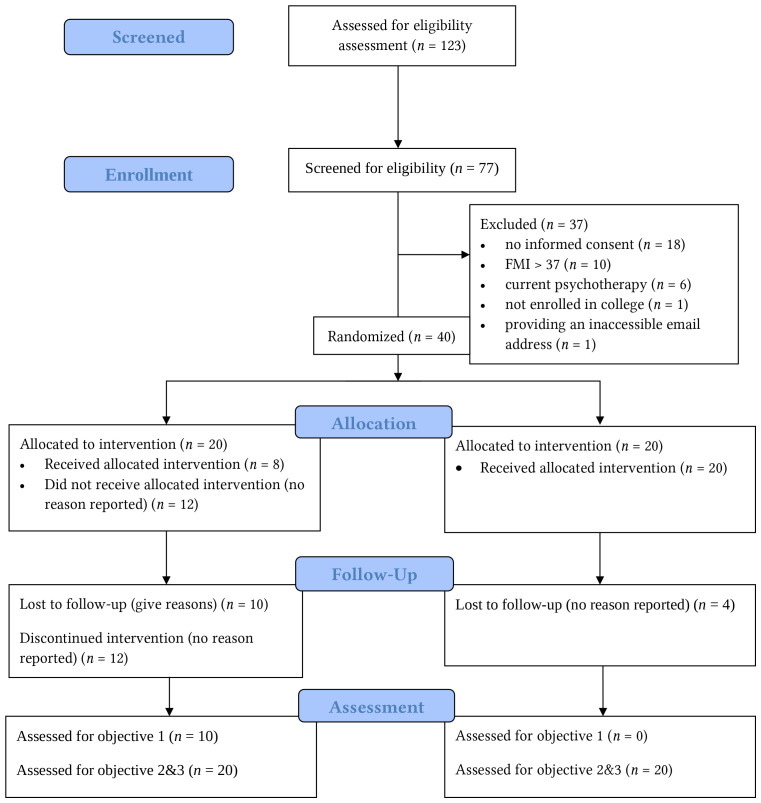
Flow Diagram

Recruitment lasted from May 2019 until March 2020. One hundred and twenty-three participants were invited to the screening. *n* = 46 did not complete the screening. Out of 77 screened, 37 were excluded due to the following reasons: not providing informed consent (*n* = 18), having a high FMI score (> 37) (*n* = 10), being in psychotherapy (*n* = 6), being in another mindfulness training (*n* = 1), not being a student (*n* = 1), and providing an inaccessible email address (*n* = 1). *n* = 40 provided consent and were randomized to either IG or WL groups, see Figure 1.

The mean age of the participants was *M* = 26.23 (*SD* = 4.51), 77.5% were female, 37.5% could speak the host country’s language well (>B2 level), and 97% speak English well (>B2 level). The study level of the participants varied: out of 40, 24 studied in a master's program, nine were in a bachelor's program, six were in a Ph.D. program, and one participant was doing an internship semester. The baseline characteristics of the participants are tabulated in Table 3.

**Table 3:** Baseline Characteristics

Variable	All Participants (*n* = 40)		IG (*n* = 20)		WL (*n* = 20)	
	*n*	%	*n*	%	*n*	%
Sociodemographic characteristics						
**Age (*M*, *SD*)**	26.23	4.5	25.05	3.5	27.40	5.2
**Female gender**	31	77.5	19	95	12	60
**Single**	23	57.5	12	60	11	55
**Knowledge of host country language** **(> B2 level)**	15	37.5	5	25.0	10	50.0
**Country of origin** Albania (*n* = 2), Belarus (*n* = 3), Belgium (*n* = 1), Cameroon (*n* = 1), Canada (*n* = 3), Colombia (*n* = 2), Costa Rica (*n* = 1), France (*n* = 2), German (*n* = 1), Ghana (*n* = 1), India (*n* = 1), Indonesia (*n* = 2), Italy (*n* = 3), Kazakhstan (*n* = 1), Kyrgyz Republic (*n* = 1), Mexica (*n* = 2), Nepal (*n* = 1), Pakistan (*n* = 1), Portugal (*n* = 1), Romania (*n* = 1), Russia (*n* = 1), Sweden (*n* = 1), Turkey (*n* = 4), Ukraine (*n* = 1), USA (*n* = 2)						
Study characteristics						
**Full-time student**	34	85	18	90	16	80
**Semester (*M*, *SD*)**	10.14	6.8	9.21	5.02	11.06	8.36
Study subject						
Business and Finance	8	20.0	4	20.0	4	20.0
Social Sciences	8	20.0	6	30.0	2	10.0
Engineering	7	17.5	4	20.0	3	15.0
Medicine & Health	5	12.5	3	15.0	2	10.0
Nature Sciences	5	12.5	0	0	5	12.5
Computer Sciences	4	10.0	1	5.0	3	15.0
Design	2	5.0	1	5.0	1	5.0
Psychology	1	2.5	1	5.0	0	0
Treatment utilization						
**Psychotherapy experience**	10	25	7	35	3	15
	*M*	*SD*	*M*	*SD*	*M*	*SD*
Outcome measures						
**Mindfulness level**	27.28	5.75	27.30	6.27	27.25	5.34
**Depressive symptoms**	16.68	3.39	18.10	2.28	19.25	3.9
**Anxiety symptoms**	17.27	4.42	16.75	4.09	17.80	4.77
**Presenteeism level**	27.85	2.21	27.8	2.40	27.9	2.05
**Well-being**	35.20	17.09	37.60	17.25	32.80	17
**Stress level**	13.38	2.44	13.10	2.31	13.65	2.58

*Note. M* = Mean; *SD* = Standard Deviation; *IG* = Intervention Group; *WL* = Waitlist control group.

Out of 40 randomized participants, 26 (IG: 50%; WL: 80%) completed the t1, resulting in a study dropout of 35%. There was a baseline difference between assessment dropouts and non-dropouts, where non-dropouts had slightly more stress (mean difference = 1.68).

#### Intervention Adherence

**Figure 2:**
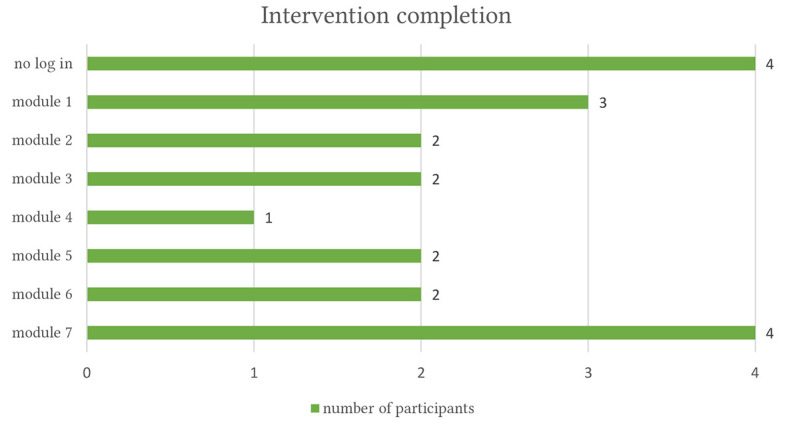
Intervention Completion

Out of 20 participants randomized into the IG, eight participants (40%) completed at least five core modules (four of them completed the seven modules), whereas four did not finish the first module. Three completed the first module, two participants completed two modules, two participants three modules and one participant completed the fourth module, see Figure 2. All the intervention completers also completed the post-randomization assessment. No reasons were reported regarding no uptake of the intervention. The average intervention duration among the intervention completers was 60 days, five of them completed the intervention within 60 days. Eight participants signed up for the SMS coach. Based on 10 participants’ answers to the open-ended questions on t1, participants practiced mindfulness on average 3.6 days weekly during the intervention. On these days, they spent an average of 18.3 minutes practicing mindfulness.

#### Acceptability

In order to assess the acceptability of the StudiCareM-E among the participants, we used various sources: open-ended questions by the end of the post-intervention measurement, treatment satisfaction measured via CSQ, and potential negative effects measured with INEP-On, and formative user feedback extracted via the online platform of Minddistrict.

According to the data from the open-ended questions at t1 (*n* = 10), five participants (25%) signed up for the SMS coach and found this helpful. Six participants stated that mindfulness meditation exercises were the most helpful element of the intervention. Body scan and body-related exercises, e.g. mindful yoga, were well-liked by the participants. Two participants stressed that example characters and the quiz on stress sources were beneficial. The majority of the participants (79%) found the length of the modules just right. On average the participants scored the feasibility of doing the modules with daily tasks 7.3 out of a 10-point scale (0 = not feasible; 10 = very feasible) and scored 3.8 on the same scale regarding the disturbance the processing of modules caused in their everyday life. Additionally, they scored 8.9 on their likelihood of participating in a mindfulness-based intervention in the future.

In terms of treatment satisfaction, the ITT data on CSQ, the overall satisfaction with the intervention was *M* = 25.4, *SD* = 2.2. All of the completers would definitely or probably recommend the intervention to a friend and 90% reported that the intervention met their needs, 70% would like to receive such intervention if they need help in the future, and 80% found the intervention satisfactory.

Potential negative effects of StudiCareM-E were evaluated with INEP-On in t1. Based on the results from INEP-On, six IG participants reported seven negative effects caused by the IMI in the following domains: anxiety about finding insurance (*n* = 1), increased financial worries (*n* = 1), data security (*n* = 1), feeling forced to do the exercises of the intervention despite not wanting to do it (*n* = 3), difficulties in making important decisions without asking the therapist (*n* = 2), found training or the formulations of the e-Coach contained hurtful statements (*n* = 1) and feeling that being made fun of in the intervention material (*n* = 1). One participant reported negative effects on each of the above-mentioned domains, whereas the rest of the five participants reported negative effects on a single domain. Of the five, two reported feeling forced into finishing modules, and three reported neglecting hobby/social contacts. No suicidal ideation was reported caused by the IMI. The magnitude of all negative effects reported was low to moderate.

According to the formative feedback extracted from the Minddistrict platform, all of the modules were well-liked, scoring ≥ 7 out of a 10-point scale, the most liked being the last module (Module 7: Body and Mind). Recommendations included adding more video/audio files, diversifying example characters’ experiences, adding more mindfulness meditation exercises, and decreasing the number of text fields.

### Efficacy Outcomes

Descriptive statistics of the study outcomes at the baseline are represented in Table 3. There were no baseline differences observed. Controlling for baseline mindfulness levels, IG participants showed improvement in mindfulness at the T1 compared to WL (β = 0.34, 95% CI [0.06, 0.63], *p* < .05; Adjusted *R*^2^ = 0.13). Moreover, anxiety was improved among IG participants, compared to WL (β = -0.42, 95% CI [-0.72, -0.11], *p* < .05; Adjusted *R*^2^ = 0.14) as well as Well-being (β = 0.37, 95% CI [0.07, 0.68], *p* < .05; Adjusted *R*^2^ = 0.13). The effect estimates (β, CI, and *p* values) of the rest of the secondary outcomes are presented in Table 4.

**Table 4:** Post-Randomization Between-Group Differences Adjusted for Baseline Values

Outcome	Baseline (T1) *M* (*SD*)	Post-treatment (T2) *M* (*SD*)	Standardized coefficient ß	95% CI	*p*
Mindfulness (FMI)	27.27 (5.75)	31.79 (4.50)	0.34	[0.06 - 0.63]	.01
Depression symptoms (PHQ-8)	18.68 (3.39)	16.89 (3.88)	-0.10	[-0.39 - 0.21]	.52
Anxiety symptoms (GAD-7)	17.27 (4.42)	15.53 (3.84)	-0.42	[-0.72 - -0.11]	.01
Stress level (PSS-4)	13.38 (2.44)	11.79 (2.05)	-0.14	[-0.46 - 0.17]	.37
Wellbeing (WHO-5)	35.20 (17.09)	44.42 (15.44)	0.37	[0.07 - 0.68]	.02
PSS (Presenteeism-Work Impairment score)	13.38 (2.44)	27.66 (1.47)	-0.01	[-0.34 - 0.32]	.94

*Note. M* = mean; *SD* = Standard deviation; FMI = Freiburg Mindfulness Inventory; GAD-7 = Generalized Anxiety Disorder Questionnaire; PHQ-8 = Patient Health Questionnaire; PSS = Presenteeism Scale for Students; PSS-4 = Short Form Perceived Stress Scale; WHO-5 = World Health Organization Well-Being Index.

## Discussion

This RCT evaluated the feasibility, acceptability, and potential efficacy of a cross-cultural version of a mindfulness-based IMI among international university students studying in Germany, Austria, and Switzerland. The initial results suggest that the adapted version of StudiCareM-E was feasible, perceived acceptable, and offered benefits in psychological outcomes compared to WL, and minor negative effects were reported among IG participants. Our preliminary results might guide a powered definitive trial. Working examples and recommendations for improvement are presented in the following paragraphs.

Our recruitment strategy included sending emails via cooperating universities, using social media channels of university groups/student clubs, and hanging hard copy posters around the Ulm University campus. We aimed at reaching a total of 40 participants, which took 11 months. The length of the recruitment is longer than a previous digital sleep intervention for international students, where *n* = 81 was reached in seven months (Spanhel, Burdach, et al., 2021). One reason for this might be the length and transdiagnostic nature of our intervention. Moreover, international student offices could be better utilized to aid the recruitment process in a future trial. With the above-mentioned strategy, we reached a population of mostly female (77%) participants, aiming for a post-graduate degree (82.5%), e.g. master's and Ph.D., which was higher than DAAD’s 2019/20 report of international students studying for a postgraduate degree in Germany (52%) (DAAD, 2020).

A post-randomization assessment dropout rate of 35% was detected. Half of the IG and 20% of the WL failed to do the post-randomization assessment. This rate is in accordance with previous mindfulness IMI among students (Lahtinen et al., 2023). It is no surprise to have fewer dropouts in a waitlist control condition because the participants of this condition got access to the intervention only after completing the post-randomization assessment. In order to avoid dropouts, we sent out six reminder emails to participants who did not complete this assessment. However, the success of these measures was limited. Future trials might include reminder SMS or phone calls to decrease the dropout rate.

The intervention adherence rate among IG participants was 40%. This rate is in line with a recent meta-analysis of online mindfulness interventions conducted with students and non-student populations, in which adherence rates ranged from 35 to 92% (Sommers-Spijkerman et al., 2021). Although guided IMI correlated with higher rates of adherence (Treanor et al., 2021; Zarski et al., 2016), this was not the case in our trial. According to a review, some factors related to an increase in adherence to IMI are the female gender, being in the control group, having time flexibility to do the intervention, computer literacy, guidance, and depth of personalized feedback to increase self-efficacy (Beatty & Binnion, 2016). Although our sample embodied some of these factors, e.g. guidance, others could be improved. Program content seems to be a decisive factor in adherence. Credibility, positive perceptions of the intervention content, personalization of the intervention team (e.g. providing a photo of the team), and intensity (e.g. too long/short and/or being too generic) of the content play a role in adherence (Beatty & Binnion, 2016). The inclusion of some persuasive design aspects might aid adherence as well (Baumeister et al., 2019). As mentioned by the participants as well, computer-human dialogue support, e.g. audio and visual content, and social support, e.g. competition, categories can be improved in a future definitive trial.

One specific component of this trial was that we adapted our intervention to a culturally diverse group of international students. This diversity of the target group might require novel intervention features beyond surface structure changes (Resnicow et al., 2000) to increase adherence. Adapting an intervention for a group of participants from various cultural, social, and financial backgrounds is particularly challenging, and naturally, offering intervention content as common as possible to be able to appeal to the majority is demanding. Therefore, one should carefully inspect all the parameters and make sure that the cultural adaptation of the IMI adds a substantial benefit to its target group. In this context, evidence of cultural adaptations’ substantial benefits is still inconclusive. Based on a recent meta-analysis, cultural adaptation of health promotion IMI might not be worth the considerable amount of effort because such adaptions do not seem to yield better effectiveness compared to active and passive controls (Balci et al., 2022). However, a previous review suggested that culturally adapted face-to-face and online interventions resulted in reducing depression and anxiety (Harper Shehadeh et al., 2016). Moreover, cultural adaptions are poorly reported in existing literature, which makes it difficult to compare across studies and draw definitive conclusions (Balci et al., 2022). The next step should include comparing an adapted IMI to a non-adapted intervention. Such dismantling trials could provide insights into whether cultural adaptation processes are actually beneficial. In a recent trial, a non-culturally adapted sleep IMI yielded beneficial effects for culturally diverse international student groups (Spanhel, Burdach, et al., 2021). This might bring out the idea that some intervention contents might not significantly benefit from an elaborate adaptation process, especially for low threshold interventions (Böttche et al., 2021; Cuijpers et al., 2018; Spanhel, Burdach, et al., 2021). This trend emerged in our results as well, where we only realized surface-level adaptations (Resnicow et al., 2000) and still found potential effectiveness. More importantly, IMI have different mechanisms of change, therefore a detailed cultural adaptation might be beneficial for a certain IMI content or delivery, but not for all (Domhardt et al., 2021; Heim & Kohrt, 2019). In a review, most of the culturally adapted interventions did not modify their core contents but included core additions and delivery methods to make the intervention more acceptable to the new target group while ensuring the fidelity of the original intervention (Chu & Leino, 2017). For mindfulness-based IMI, valued living, cognitive fusion, present moment awareness, and acceptance are effective mediators among college students (Levin, Haeger, Pierce, & Twohig, 2017; Viskovich & Pakenham, 2020). Some of these mediators are part of the universal human condition, therefore, might not even need any adaptation. Lastly, acculturation might play a role in attitudes toward seeking mental health (Lu et al., 2014). Therefore, acculturation levels of international students might be considered when adapting or developing interventions for this population.

Only six negative effects were reported and these were low to mild in extent. Moreover, the IMI caused no suicidal ideation. Negative effects of psychotherapy are expected and their reporting is increasing (Rozental et al., 2018). This result suggested that StudiCareM-E is a rather safe intervention, and might be also administered in an unguided form.

Furthermore, StudiCareM-E participants showed improvements in mindfulness, anxiety, and well-being levels. Stress and depression scores did not reach significance. While a trend suggests possible beneficial effects regarding these outcomes, a powered definitive trial would be necessary to confirm these effects since this trial was only powered for feasibility. The mean effect sizes are higher than in a meta-analysis of online mindfulness interventions compared to a waitlist and no-treatment controls (Spijkerman et al., 2016). However, trials with waitlist control groups tend to yield higher effect sizes (van Agteren et al., 2021), thus in order to validate the StudiCareM-E’s efficacy, research should initially test this in a powered trial with more follow-up points, and compare it to treatment as usual, a placebo control group or active controls.

Like any other, this trial is not free from limitations. Firstly, our sample mostly consisted of female participants, therefore our results cannot be generalized to male or non-binary populations. However, this is a common trend in psychological interventions. Secondly, a major limitation of this trial was grouping international students from various backgrounds and living situations under the label of international students, consequently masking potential differences among them. Thirdly, our sample consisted of participants with diverse cultural backgrounds. According to a meta-analysis of 99 studies, it was found that studies with more homogenous participants in terms of cultural background yielded larger effect sizes (Soto et al., 2018). Even though culturally adapted, this intervention was in English. People prefer to have a unity of language with their mental health care provider (Villalobos et al., 2016), and providing interventions in the chosen language of the client is a significant predictor of better outcomes (Soto et al., 2018). Despite this fact, participants assessed the language of the intervention as being easy to understand. However, still providing the intervention content in the participant’s chosen language might increase the efficacy of the intervention further. Therefore, a future definitive trial might consider offering the same intervention in different languages to choose from and might adapt the intervention based on parsimonious social and cultural features. Fourth, this feasibility trial used a WL control group. As expected, trials of culturally adapted face-to-face mental health interventions with a WL group resulted in higher effect sizes, compared to an active control condition (*d* = 0.53 vs *d* = 0.47) (Soto et al., 2018). This is also true for IMI (Sommers-Spijkerman et al., 2021). Fifth, due to high dropout and low adherence, we were able to collect less qualitative and quantitative data to inform acceptability and potential efficacy. Assessment dropout was 35% in total, which is in accordance with the previous research (Nilsson et al., 2004). Possible reasons for this may include a lack of monetary incentives, procrastination, and the typical workload of student life. In order to tackle potential bias arising from differential dropout, we multiply imputed our data with the assumption of missing at random (Bell et al., 2013), and added baseline values as covariates in all regression models. However, there was a baseline difference between assessment dropout and non-dropouts where, participants who completed the post-randomization assessment had a slightly higher stress level in the beginning of the study, therefore might be more motivated, needed a medium to deal with the stress, and had more place to grow. Lastly, this feasibility trial reached a limited sample size; therefore, the initial efficacy results should be interpreted with caution. An inspection of sustainability of intervention effect beyond post-treatment is warranted.

### Conclusion

Online interventions to decrease stress and improve the well-being of international university students seem to have great potential, whereas face-to-face offers are not often utilized and benefited in limitation. Despite being presented to vastly culturally diverse student groups, StudiCareM-E yielded beneficial results with good acceptability and non-crucial negative effects. A future definitive RCT might offer a more robust efficacy and potential moderator and mediator effects.

## Supplementary Materials

The Supplementary Materials contain the pre-registration information for the study (for access see Index of Supplementary Materials below).



BalciS.
KüchlerA.
EbertD. D.
BaumeisterH.
 (2019). English version of the StudiCare mindfulness: A randomized controlled pilot study
[Pre-registration protocol; DRKS-ID: DRKS00017507]. PsychOpen. https://drks.de/search/en/trial/DRKS00017507


## Data Availability

The dataset may be obtained (from S.B.) on request depending on to-be-specified data security and data exchange regulation agreements. To ensure confidentiality, shared data will exclude any identifying participant information.

## Reporting Guidelines

This article follows the guidelines of CONSORT statement.
